# Sexual dysfunction and health condition in Chinese doctor: prevalence and risk factors

**DOI:** 10.1038/s41598-020-72072-w

**Published:** 2020-09-16

**Authors:** Weiran Li, Shixing Li, Pengfei Lu, Haibin Chen, Yunyu Zhang, Yu Cao, Guanjian Li

**Affiliations:** 1 Institute of Dermatology and Department of Dermatology, the First Affiliated Hospital, Anhui Medical University, Hefei, Anhui China; 2 Key Laboratory of Dermatology, Anhui Medical University, Ministry of Education, Hefei, Anhui China; 3 Norman Bethune Health Science Center of Jilin University, Changchun, Jilin China; 4 China-Japan Union Hospital of Jilin University, Changchun, Jilin China; 5 The First Affiliated Hospital of Xingjiang Medical University, Wulumuqi, Xingjiang China; 6 Xingjiang Medical University, Wulumuqi, Xingjiang China; 7 Jiangsu Province Hospital, Nanjing, Jiangsu China; 8 The First Affiliated Hospital With Jiangsu Medical University, Nanjing, Jiangsu China; 9 The Second Affiliated Hospital of Guangdong Medical University, Guangzhou, Guangdong China; 10 The First Affiliated Hospital of Anhui Medical University, Hefei, Anhui China; 11 Anhui Medical University, Hefei, Anhui China

**Keywords:** Diseases, Health care, Health occupations, Risk factors

## Abstract

To our knowledge, little attention has been paid to the sexual function of health workers. The aim of the present study was to assess the frequency of sexual dysfunction among Chinese doctors. In addition, the risk factors for sexual dysfunction were analyzed. This was a questionnaire-based multicenter cross-sectional survey performed in five large academic medical centers in China. A total of 539 male doctors, 547 female doctors were evaluated. We analyzed doctors' demographic characteristics, quality of life, sexual function and attitudes towards sexual problems. Chinese doctors are at high risk of sexual dysfunction and poor health. The prevalence of sexual dysfunction appears to increase with age, and is associated with various social and demographic factors including monthly income, physical exercise, working hours, night shift and health-related quality. The quality of life and sexual health of doctors deserves special attention as a significant public health concern. Alleviating work pressure, increasing income, improving quality of life and therapeutic sexual problems should be considered comprehensively.

## Introduction

Sexual functioning is an important part of human lives that relates to mental and physical wellbeing and social relations^1–3^. Sexual problems and dysfunctions are commonly encountered experiences that impact on the quality of life in the general population^4,5^.

According to numerous studies, sexual dysfunctions are highly prevalent in both sexes, ranging from 10 to 52% of men and 25% to 63% of women^6^. The large discrepancy in prevalence across studies could be attributed to the differences in the populations, the sampling methods, diagnostic criteria and assessment tools^7–9^. Among men, the most prevalent problems were erectile dysfunction, premature ejaculation, and orgasm problems. Whereas among women, the most frequent were low desire, reduced arousal, difficult to achieve an orgasm, and dyspareunia^10,11^. The reasons for sexual dysfunction are multiple and complex. In recent years, epidemiological researches have found associations between sexual dysfunction and many personal characteristics, such as age, education level, marital status, household income, mental and physical health problems^12^. Currently, sexual dysfunction have been studied extensively in different populations, but the sexual function of health workers has received very little attention^13,14^. In fact, these scanty studies only focused on the risk factors of sexual dysfunction, such as age and related diseases. That is, in these studies, the health worker groups was considered only as readily accessible samples, their sexual function has not been specially analyzed.

In general, doctors are considered to have a higher degree, higher incomes, and better health care than most of their peers. It is noteworthy that these characteristics have also been widely described in association with sexual dysfunction. On the other hand, numerous studies have demonstrated a clear relation between sexual function and quality of life^15,16^. As a particular occupational population, doctors undertake the mission of curing patients and exposure to disease, death, emergency which is believed to lead to a high level of occupational stress. Additionally, stress from night shift work, overtime, and other occupational characteristics, such as chemical exposure, radiation sources, musculoskeletal strain and disorders can also affect the mental and physical health of doctors^17–19^.

Thus, it is necessary to examine the patterns, demographic and clinical correlates of sexual dysfunction in doctors. Knowledge about doctors’ sexual functioning as well as early detection of sexual problems and dysfunctions is essential to promote a healthy sexual lifestyle which has unintelligible influence on their mental and physical health. And, furthermore, it may has implications for stability in doctors’ workforce and the quality of health cares it provides.

China is a country with a long history of conservative and traditional beliefs, thus the Chinese, even the Chinese doctors, are reluctant to discuss sexual matters openly^20^. The cultural factors make investigations of sexual function difficult. Actually, there have been no published studies in journals that evaluate the sexual function of Chinese doctors as far as we know. However, with the rapid advancement of internet technique and service, online surveys may provide a helpful solution for this case. China has the most internet users in the world, at 802 million in 2018. The internet service has become an essential open conduit for business, learning and daily communication in this country. Several studies have demonstrated that performance and effectiveness obtained using Internet surveys on sexual problems concur with those obtained using other methods, such as mailing questionnaires or telephone surveys^21–23^. What's more, online surveys may even provide a more relaxed and private environment, while allowing convenient data collection and analysis.

Based on such considerations, this survey was designed to provide the frequency of sexual dysfunction in Chinese doctors and to determine sociodemographic factors associated with clinically problems.

## Methods

### Participants

This multicenter cross-sectional survey was performed in five large academic medical centers in China from May to July 2019.

By using cluster sampling method, a population consisted initially of 1,396 doctors registered and working in five medical centers. These five centers located in the east, west, south, north, and centre of China respectively, can be considered as representative samples of this country's major public hospital. In considering the feasibility and convenience, a nonprobability sampling method was used in this survey. The final participants came from different department including internal medicine, surgery, pediatrics, obstetrics and gynecology, stomatology.

Informed consent was furnished according to the Declaration of Helsinki and obtained from all subjects after they were provided a detailed description of the experimental procedures and informed that they could withdraw from the survey at any time. All experimental protocol in this study involving human participants was approved by the Ethic Committee of Anhui Medical University and the relevant hospitals. (Approval number: PJ20190707; Clinical trial registration no. ChiCTR1800020321). We organized a multidisciplinary team of health professionals, including health and counselling psychologists, network engineers, venereologists, gynecologists, and reproductive biologists. We are confirming that all experiments were performed in accordance with relevant guidelines and regulations that promote respect for all human subjects and protect their health and rights. All study participants approved an electronic copy of informed consent to indicate their agreement for the clinical data to be used in scientific research and publication. Before the questionnaire was distributed, five graduate students and three professors participated in a pilot study to verify and modify the contents of the questionnaire.

A web page link to our questionnaire was sent to the doctors during breaks (https://www.wjx.cn/jq/83291804.aspx). The questionnaire system avoids duplicate entries by preventing users with the same IP address access to the survey twice. Respondents can able to choose an appropriate time to fill out the questionnaire (not busy or in a private environment). This survey was anonymous and participants' information was strictly confidential. The current questionnaires were self-administered and each subject was paid about $5 for participating in this survey.

A total of 1,396 participants (681 men, 715 women) were invited in this survey and 1,278 doctors (615 men, 663 women) submitted the questionnaire, overall participation rates of 90.3% for men and 92.7% for women. The sample size was based on a pilot survey of 160 doctors done in Hefei, Anhui, China in November, 2018. Based on the assumptions (two-tailed α of 0.05, power of 90.0%), a minimum of 897 participants was needed. In order to enhance the credibility of the answers to the questions on sexual function, subjects who had been sexually active, meaning having sex at least once in the last 6 months were included. Among the 1,278 participants, 192 were excluded for the following reasons: pregnant (n = 36); hormone replace-ment therapy (n = 8); diabetes, heart disease and malignant tumors (n = 75); not meet the sexually active criterion (n = 73). Ultimately, 1,086 valid questionnaires were used in the data analysis.

The survey started with an initial interface explained the purpose of the research, obtained informed consent and reviewed the patients’ answers regarding exclusion criteria. In order to enhance the quality of this research, a missing answer reminder component was appended in the system to avoid data missing and ensure data integrality. In addition, an experienced medical expert was available to help respondents with any survey difficulties on the internet or by telephone.

### Each parts of the questionnaire

Section 1: focused on the socioeconomic and demographic status of respondents, such as age, gender, height, weight, marital status, education levels, history of diseases, monthly income, physical exercise status, whether menopause, reproductive history, work hours and night shift work situations, etc. These specific categories under each variable are shown in Table 1.

**Table 1:** Prevalence of sexual dysfunction items by demographic characteristics (n = 1,086).

Variables	No	Sexual dysfunction		χ^2^	*P*
		Yes	No		
**Sex**					
Male	539	109 (20.2)	430 (79.8)	103.760	0.001
Female	547	272 (49.7)	275 (50.3)		
**Age (years)**					
21 ~	384	134 (34.9)	290 (65.1)	9.106	0.011
35 ~	579	190 (32.8)	349 (67.2)		
≥ 50	123	57 (46.3)	66 (53.7)		
**BMI (kg/m**^**2**^)					
< 18.5	30	10 (33.3)	20 (66.7)	1.776	0.620
18.5–24	501	185 (36.9)	316 (63.1)		
24–28	444	146 (32.9)	298 (67.1)		
≥ 28	111	40 (36.0)	71 (64.0)		
**Education level**					
College or below	37	14 (37.8)	23 (62.2)	0.132	0.936
Bachelor	141	49 (34.8)	92 (65.2)		
Master or above	908	318 (35.0)	590 (65.0)		
**Marital status**					
Married	876	298 (34.0)	578 (66.0)	2.254	0.133
Unmarried	210	83 (39.5)	127 (60.5)		
**Monthly income (yuan)**					
< 5000	168	73 (43.5)	93 (56.5)	13.608	0.001
5000–10,000	456	172 (37.7)	284 (62.3)		
> 10,000	462	136 (29.4)	326 (70.6)		
**Physical exercise (per week)**					
< 1 time	534	223 (41.8)	311 (51.2)	22.065	0.001
1–2 times	273	85 (31.1)	188 (68.9)		
≥ 3times	279	73 (26.2)	206 (73.8)		
**Night shift**					
Yes	739	298 (40.3)	441 (59.7)	27.904	0.001
No	347	83 (23.9)	264 (76.1)		
**Working hours (per week)**					
< 40 h	47	16 (34.0)	31 (66.0)	10.589	0.014
40–47 h	270	83 (30.1)	187 (69.3)		
48–55 h	446	132 (29.6)	314 (70.4)		
≥ 56 h	323	130 (40.2)	193 (59.8)		
**Have child (only female)**					
Yes	429	212 (49.4)	217 (50.6)	0.076	0.783
No	118	60 (50.8)	58 (49.2)		
**Menopause (only female)**					
Yes	71	57 (80.3)	14 (19.7)	30.472	0.001
No	476	215 (45.2)	261 (54.8)		

Section 2: assessed sexual function. Sexual function was evaluated with the Arizona Sexual Experience Scale (ASEX) in this survey. It is an effective, short, easy to use, reliable, non-invasive and unisex instruments to assess sexual functioning^24–26^. The translated version of the ASEX contains 5 items, providing a total score ranging from 5 and 30, items on the ASEX query sexual drive, ease of sexual arousal, penile erection or vaginal lubrication, ability to reach orgasm and satisfaction with orgasm in the past week^27^. Sexual dysfunction was defined as a total score 19 or more, any item 5 or more, or any three items 4 or more. This scale achieved good internal consistency, excellent test–retest reliability (two weeks, r = 0.80 and 0.89), and good construct validity. The Chinese version of the ASEX has been subsequently validated with different clinical samples, and was concluded that this version has high validity and reliability^28,29^.

Section 3: assessed quality of life. Health-related quality of life was evaluated with the validated Chinese version of the Short-Form 12. This section has twelve items and is composed of eight domains: physical functioning (PF), role limitations due to physical functioning (RP), bodily pain (BP), general health perceptions (GH), vitality (VT), social functioning (SF), role limitations due to emotional problems (RE), and mental health (MH). In general, the physical component summary (PCS) includes PF, RP, BP, and GH, and mental component summary (MCS) includes VT, SF, RE, and MH. Dimension score is converted into a standard 100 scores, a higher score indicates a better quality of life^30,31^. In fact, as a short, generic measure of QOL, Short-Form 12 has been proved to have high applicability and reliability for the Chinese samples^32–35^.

Section 4: an examination of help-seeking behavior. In this section, two questions were asked: First, do you need professional guidance on sexual function? Second, have you received any medical advice or treatment for sexual problems?

At the end of this questionnaire, participants were invited to provide an e-mail address in order to know the final results of this study, as well as for the possible further observational follow-up study.

### Statistical analysis

SPSS for Windows (SPSS 18.0, Chicago, IL, USA) were used for statistical analysis, with a significance level of 5% (double tailed). chi-square tests, Student’s t-test, regression analysis model were used to describe the relationships among the variables under suitable conditions. The database was established using EpiData V3.1 software. Bartlett’s test and Kaiser Meyer Olkin measure were used to evaluate the validity. The reliability were evaluated by Cronbach’s α coefficient. Forward stepwised logistic regression was used to identify risk factors related to with sexual dysfunction.

## Results

Ultimately, a total of 539 male doctors, 547 female doctors aged 24 to 61 (37.12 ± 10.3) years old were evaluated.

Table 1 displays the basic demographic characteristics of the population. In this survey, about 19.3% of the participants were unmarried, and 80.7% were married. Only 11.3% of the respondents were 50 or older and more than half (83.6%) of them had a master degree or higher. In addition, nearly 95.7 percent of doctors worked more than 40 h a week, 42.5% of them had monthly incomes above 10,000 yuan (approximately $1,454 in 2019).

### Sexual dysfunction in male and female doctors

Table 2 shows the mean scores of sexual drive, sexual arousal, lubrication/erection, orgasm and sexual satisfaction of the subjects. Built on the self-reported, the prevalence of sexual dysfunction was 35.1% in the whole sample, 20.2% in males and 49.73% in females. Female doctors report significantly higher rates of sexual dysfunction than male doctors (χ^2^ = 103.760, *P* = 0.001). Specifically, female doctors were more likely to have sexual drive, sexual arousal and orgasm problems than male.

**Table 2:** ASEX scores of the subjects (n = 1,086).

	Male		Female	
	Function (n = 430) Mean (SD)	Dysfunction (n = 109) Mean (SD)	Function (n = 275) Mean (SD)	Dysfunction (n = 272) Mean (SD)
Total ASEX score	13.32 (2.47)	22.93 (3.01)	14.86 (2.38)	23.57 (3.46)
Sexual drive	2.27 (0.93)	4.42 (1.13)	3.26 (0.92)	4.85 (0.97)
Sexual arousal	2.34 (0.69)	4.45 (0.83)	2.45 (0.74)	4.56 (1.02)
lubrication/erection	3.13 (0.78)	4.81 (1.25)	3.04 (0.84)	4.80 (1.17)
Orgasm	2.74 (0.80)	4.77 (1.08)	3.33 (0.95)	4.91 (0.92)
Sexual satisfaction	2.84 (0.85)	4.48 (0.91)	2.78 (0.83)	4.45 (1.04)

*ASEX* Arizona Sexual Experience Inventory Scale, *SD* standard deviation.

**Figure 1:**
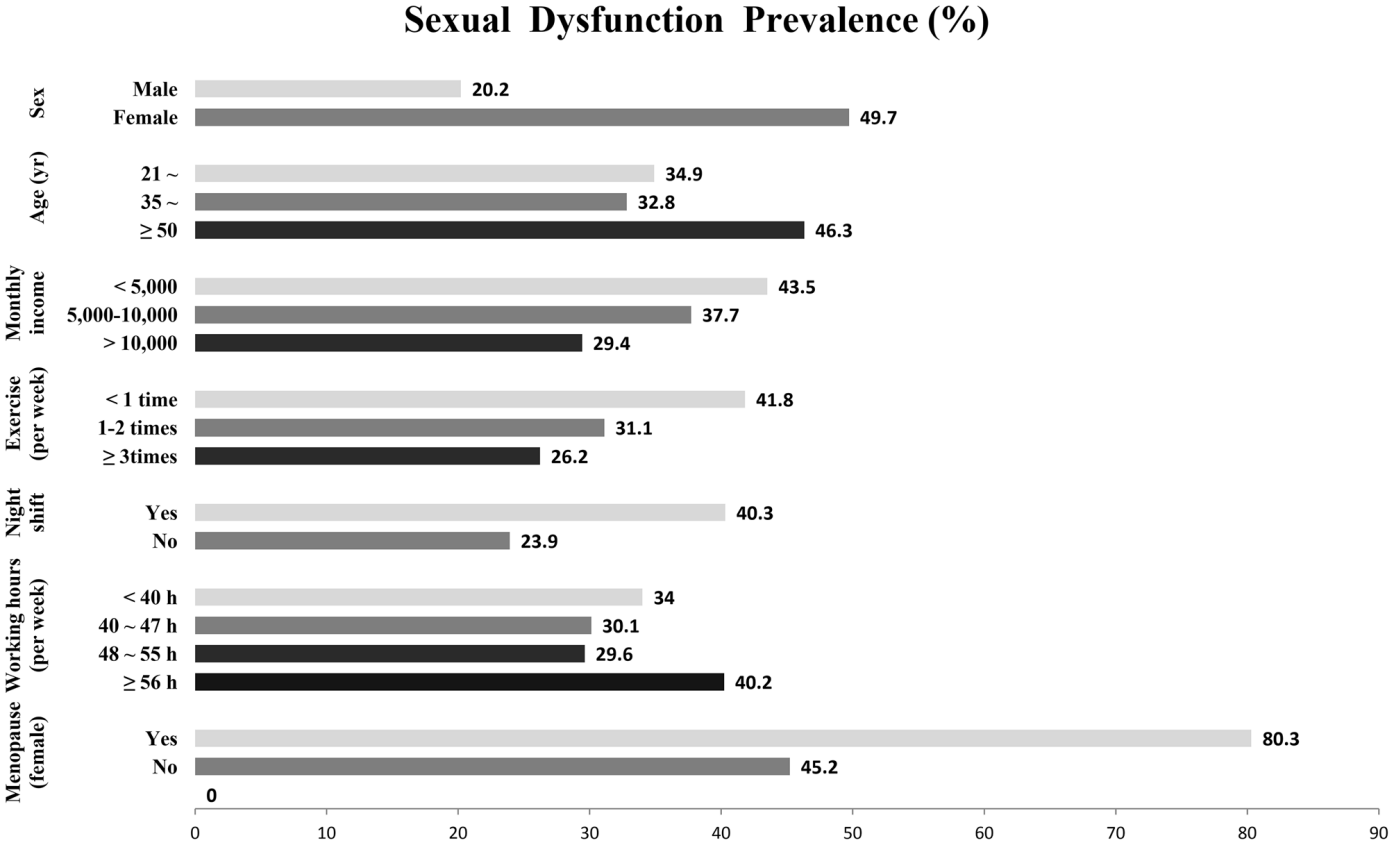
Sexual dysfunction prevalence in different groups.

Differences in the incidence of sexual dysfunction between groups were presented in Fig. 1. Compared with doctors aged 21–34 years, the prevalence of sexual dysfunction among doctors aged 50 years or older was higher. In addition, more night shifts, longer working hours, lower monthly income, less physical exercise were strongly associated with increased risk for sexual dysfunction. The prevalence of postmenopausal female subjects was significantly greater than that of premenopausal female subjects. However, there was no significant association between the sexual dysfunction and marital status or education level in this research.

### Health-related quality of subjects

Table 3 shows the features of the total and dimension scores of the health-related quality. The mean score of PCS and MCS was 43.2, 41.7 for male and 43.1, 40.3 for female, respectively. It is worth reminding that all these values were significantly lower than the midpoint of the total scales (50), suggesting that the health status of these doctors were poor.

**Table 3:** Health-related quality of the subjects (n = 1,086).

	Male		Female	
	PCS	MCS	PCS	MCS
Mean (SD)	43.2 (9.1)	41.7 (7.3)	43.1 (6.4)	40.3 (7.5)*
Median	43.6	41.9	43.2	40.4
Percentile 25th	37.0	36.0	38.0	34.8
Percentile 75th	50.3	47.1	47.8	45.6

*PCS* physical component summary, *MCS* mental component summary.

### Risk factors of sexual dysfunction

Table 4 shows the demographic and clinical correlates of sexual dysfunction. A forward stepwise multiple regression analysis was used to analyze the impact of variables. After adjusting for confounding variables, we found that age (2.14, *P* = 0.009), monthly income (OR = 0.45, *P* = 0.023), physical exercise (OR = 0.57, *P* = 0.005), working hours (OR = 1.65, *P* = 0.017), PCS (OR = 0.63, *P* = 0.001), and MCS (OR = 0.66, *P* = 0.038) were identified as risk factors of sexual dysfunction in male doctors. In all female, age (OR = 1.079, *P* = 0.009), night shift (OR = 1.38, *P* = 0.040), working hours (OR = 2.15, *P* = 0.026), menopause (OR = 5.05, *P* = 0.010), PCS (OR = 0.70, *P* = 0.047), and MCS (OR = 0.43, *P* = 0.019) were significant predictors of sexual dysfunction.

**Table 4:** Correlates of sexual dysfunction among subjects as determined by logistic regression analysis (no sexual dysfunction as the reference group).

	Male				Female			
	Univariate analysis	Multivariate analysis			Univariate analysis	Multivariate analysis		
	*P*	OR	*P*	95% CI	*P*	OR	*P*	95% CI
Age (years)	0.001	2.14	0.009	1.50–3.05	0.001	2.67	0.001	1.80–3.76
BMI (kg/m^2^)	0.506	–	–	–	0.364	–	–	–
Education level	0.872	–	–	–	0.524	–	–	–
Marital status	0.355	–	–	–	0.470	–	–	–
Monthly income	0.014	0.45	0.023	0.24–0.96	0.173	–	–	–
Exercise situation	0.002	0.57	0.005	0.37–0.84	0.046	–	–	–
Night shift	0.158	–	–	–	0.013	1.38	0.040	1.04–2.36
Working hours	0.001	1.65	0.017	1.11–2.66	0.001	2.15	0.02	1.31–4.96
Have child	–	–	–	–	0.608	–	–	–
Menopause	–	–	–	–	0.002	5.05	0.010	1.82–8.75
PCS	0.001	0.63	0.001	0.44–0.95	0.025	0.70	0.047	0.52–0.94
MCS	0.012	0.66	0.038	0.48–0.92	0.008	0.43	0.019	0.20–0.89

Age, BMI, PCS and MCS were taken as continuous variables in this model, while the remaining variables were defined as categorical variables and were shown in Table 1.

*BMI* body mass index, *PCS* physical component summary, *MCS* mental component summary.

### Reliability and validity analysis

The overall Cronbach’s α coefficient of the profile was 0.810, indicating that the questionnaire had good internal reliability. Exploratory factor analysis showed that KMO measure was 0.836 and Bartlett’s sphericity *P* value was 0.001, indicating that the data collected from the instrumental were valid for factor analysis.

### Help-seeking behavior examination

The help-seeking behavior examination indicated that most of the participants (82.2%) expressed need on the guidance of sexual life. Our data on help-seeking also suggested that only 3.15% of these participants sought medical consultation for their sexual problems.

## Discussion

This was a questionnaire-based multicenter cross-sectional survey performed in five large academic medical centers in Nanjing, Hefei, Changchun, Urumqi and Zhanjiang from May to July 2019. Participants were enrolled from these five medical centers through a nonrandom cluster sampling procedure.

Our results show that the prevalence of sexual dysfunction was 49.73% in females doctors. In all ASEX items, female doctors endorsed more frequent sexual dysfunction than male doctors, which is consistent with recent studies^36,37^. In previous surveys, about 40% of female reports some form of sexual problems^38,39^, and the prevalence may be even lower in China. For instance, Ma et al. interviewed 586 married women aged 22 to 60 who visited a health clinic and reported a prevalence rate of 37.6% in China^40^. In another study, a survey showed that 37.9 percent of the young and middle-aged women in Hong Kong had sexual dysfunction^41^. Similarly, Luo et al. suggested that the incidence of sexual dysfunction in Chinese women was 26.5 percent, less than one-third^42^. Factors contributing to the diverse rates include various sociocultural backgrounds, sample selection, assessment methods, assessment procedures and different cutoff values. Recently, in a study that used a similar assessment method to ours, 18.4% of Spanish women and 28.7% of Portuguese women were classified as having sexual dysfunction^10^. In fact, this prevalence appeared to be much lower than that of Chinese female doctors in our study. Despite these demographic and methodological differences, we concluded that Chinese doctors, especially women, are at high risk of sexual dysfunction.

In the current study, the percentage of participants with sexual dysfunction generally increased with age, as showed in Fig. 1. However, the overall prevalence of sexual dysfunction among people aged 35–50 has declined slightly. Among the youngest participants, the high prevalence of sexual dysfunction may reflect the fact that a considerable proportion of young doctors are currently facing serious sexual problems. Several contributing factors may account for this phenomenon. The high incidence of sexual dysfunction among young doctors may be associated with a higher proportion of singles, less sexual experience and a higher rate of sexual partner turnover. On the other hand, tremendous work pressure, economic problems and poor physical and mental health may also play an important role in the high incidence of sexual dysfunction among young doctors in China. The correlation between these factors and sexual dysfunction has been widely reported^12,43^. Sexual problems are also common among older doctors. As some literatures have reported, they are more likely to suffer from poor lubrication or erectile dysfunction, as well as to lack an interest in sex^44,45^.

Table 3 shows that the physical and mental health of Chinese doctors in this study are very poor. In recent years, China's huge population base and growing demand for patient health have led to overworking of doctors. The proportion of doctors in the total population in China is 1:735, which is much lower than that in Western countries^46^. Numerous studies, including our research (Table 1), have reported that Chinese doctors face enormous occupational stress due to their low income, long working hours and poor working environment^47,48^. It is noteworthy that sexual dysfunction is widely suggested being associated with stress and poor quality of life^2^. Data from a large British study (NHSLS data) show that emotional and stress-related problems increase the risk of sexual difficulties. It is also suggested that physiological and psychological state are independent factors affecting sexual function^49^. What's more, another study from Denmark found that poor health is linked to the increasing incidence of sexual dysfunction in both sexes. Physical health problems mainly affect men's sexual life, and mental health problems are closely related to female sexual dysfunction^50^. Therefore, we believe that a series of effective measures, such as reducing workload and increasing income, may be helpful to reduce work pressure and improve health. We suggest that step up the quality of life of doctors should be considered in the current medical reform, which may be meaningful for improving sexual function among doctors in China.

A number of previous studies consistently reported that only a small proportion of patients with sexual dysfunction may actually seek help, ranging from 6 to 32%. In fact^51–53^, our study showed that the majority (82.2%) of doctors with sexual dysfunction reported that they need professional guidance. Surprisingly, only 12 doctors (3.15%) received any medical advice or treatment at all. That is, despite the relatively high prevalence of sexual dysfunction among sexually active doctors, we observed that few participants seek help for their problems. This may be linked to traditional Chinese beliefs that discussion of sexual issues is intensely private. With the continuous development and progress of society, Chinese are more open to discussing sexual problems. However, they are still reluctant to discuss doctors. These doctors might be unwilling to raise sexual issue with specialist not only because of shyness but also due to many doctors may feel embarrassed when reporting their diseases, especially facing colleagues^54^. We should note that doctors are always regarded as models of health knowledge and behavior by the whole society. It is of great importance to doctors and other health professionals to be aware of common sexual difficulties in themselves and patients, and manage them.

The current study was based on an online survey that is generally considered less reliable. But notice that despite the increasing openness and diversity of society, Chinese are still reluctant to discuss sexual issues openly. We believe that an anonymous online questionnaire evaluation system may be a more suitable tool which can provides a comfortable and free environment for people to report and discuss their sexual issues.

However, the causal relationship between quality of life and sexual dysfunction has yet to be investigated. As our data were cross-sectional, we can't infer causality in the relationships we show. In other words, among various factors associated with sexual dysfunction, two-way causality is an obvious possibility. Despite these confusions and concerns, many studies have shown that sexual life satisfaction, mood and quality of life were strongly correlated^2,43^. In addition, recent advances in therapy for sexual dysfunction may have beneficial effects on quality of life. Regardless of these complex situations, to solve these problems, decision-makers should strive to improve the income and working environment of doctors. On the other hand, researchers should focus on identifying the relevant factors and mechanisms of sexual problems as well as developing appropriate therapies.

Participants in this study were restricted to public hospitals in cities, so there may be better working conditions than private or rural clinics. In China, urban doctors were the most likely to have higher education, higher income and better health care than rural doctors. However, most hospitals are public institutions with fewer private clinics in China. Therefore, we initially focus on municipal hospitals, and expect to consider rural clinics in large multi-center research in the future.

In this survey, only sexually active participants (at least once in the last 6 months) were incorporated into the final analysis, so excluded individuals who may not have had sex recently. This manner may limit our conclusions as excluded respondents may have avoided sex due to sexual difficulties. Excluded doctors were more likely to be single and have lower monthly incomes. In addition, we don't have information about the sexual partners or who used drugs that could suppress libido.

We did not consider variability by ethnicity in this analysis. The Han is the majority ethnic group in China. Before formal study, we have found that the proportion of respondents from minority ethnic groups was too low for reliable analysis (1.9%) in our pre-investigation. We currently undertaking a focus study among ethnic minorities to address this question.

That is, prevalence estimates for the studied sexual dysfunctions may not be applicable to the entire physician groups, but they are most likely to be effective for the sexually active majority of Chinese doctors. Despite these limitations, this is the first report on sexual function of doctors in China.

In general, our findings noted that most Chinese doctors reported having sexual dysfunction and a poor quality of life. The prevalence of sexual dysfunction appears to increase with age, and it is associated with various social and demographic factors including monthly income, physical exercise, working hours, night shift and health-related quality.

Considering the professional characteristics and exemplary role of doctors, their risk management of life and sexual health problems deserves special attention from health policy makers as a significant public health concern.

## Data Availability

The authors declare that all data supporting the findings of this study are available within the article or from the corresponding author upon reasonable request.
